# Epidemiology of schizophrenia and its management over 8-years period using real-world data in Spain

**DOI:** 10.1186/s12888-020-02538-8

**Published:** 2020-04-05

**Authors:** A. Orrico-Sánchez, M. López-Lacort, C. Muñoz-Quiles, G. Sanfélix-Gimeno, J. Díez-Domingo

**Affiliations:** 1 Vaccine Research Unit, Fundación para el Fomento de la Investigación Sanitaria y Biomédica de la Comunitat Valenciana, FISABIO-Public Health, Valencia, Spain; 2 Health Services Research Unit, Fundación para el Fomento de la Investigación Sanitaria y Biomédica de la Comunitat Valenciana, FISABIO-Public Health, Valencia, Spain

**Keywords:** Schizophrenia, Incidence, Prevalence, Real world data, Epidemiolgy, Antipsychotics

## Abstract

**Background:**

Real-World Data (RWD) studies provide important insights in disease epidemiology, in real clinical populations, with long follow-up periods. The aim of the present study was to describe the epidemiology of schizophrenia spectrum disorders (SD) during an 8-year period in Spain.

**Methods:**

A retrospective cohort of subjects aged 15 to 64 years was followed-up using electronic healthcare databases of the Valencia region (2008–2015). SD cases included outpatient and inpatient settings (ICD 9 codes 295.XX). Prevalence of SD was assessed. Incidence rate (IR) in the subpopulation aged between 15 and 34 years was also provided. Healthcare utilization (HCU) rates, including outpatient, specialists, hospitalizations and antipsychotic dispensations were estimated.

**Results:**

The cohort included 3,976,071 subjects; 24,749 of them had a prevalent diagnosis of SD. The overall prevalence for SD was 6.2 per 1000 persons. SD were 76% more prevalent in men than women. IR in the subpopulation aged between 15 and 34 years was 50.25 per 100,000 persons years and was more than 2 times higher for men than for women. 83.4% of the overall outpatient visits from the cohort of patients were related to SD. The 21,095 overall hospitalizations with the SD code resulted in 286,139 days of hospitalization, with a median of 4 days (IQR: 1.6–9.2) per person-year. 93.2% of subjects diagnosed with SD were ever treated with some antipsychotic drug during the study period, and 70% of the patients were ever treated with antipsychotic polypharmacy.

**Conclusions:**

This large population-based study using RWD provides novel and recent information SD in a southern European country. The prevalence and IR of SD showed is greater than previously published and higher in men than in women. The fact of having used a large arsenal of electronic data (including outpatient and inpatient) for 8 years may have influenced. SD represents high burden and healthcare utilization. Contrary to guidelines recommendations the majority of patients were ever treated with antipsychotic polypharmacy.

## Background

Schizophrenia, a chronic and complex neuropsychiatric disorder, causes a significant impact on the quality of life of patients and their families, the social environment, and the healthcare system [1]. Schizophrenia spectrum disorders (SD) affects over 21 million people worldwide [2]; it has been estimated that approximately seven individuals per 1000 will develop SD during their lifetime [3], with onset of symptoms usually during the second or third decade [4, 5]. Disease occurrence may be associated with genetic, socio-demographic and environmental factors [3, 4, 6].

For decades it was thought that incidence of schizophrenia was consistent regardless of the geography and the time period. However, several systematic reviews have elucidated high variability of schizophrenia incidence rates among sites, ranging from 8 to 43 per 100,000 individuals [3, 6]. There is also high inconsistency in results among studies regarding the prevalence of schizophrenia, reporting up to 13-fold variation (from 0.12 to 1.6 per 100) [3, 7–9]. These discrepancies together with the scarcity of recent evidence on the incidence and prevalence published since 2005 [3, 7, 9] highlight the need for epidemiological population-based high quality studies providing incidence and prevalence estimates for different geographical settings and time periods.

Understanding resource consumption of patients with SD is a cornerstone to know the global burden of this disorder. Several studies have estimated the consumption of resources by antipsychotic treatment [10–13]. However, there is scarcity on the overall burden from a population-based cohort of subjects with SD, and these estimates are considered necessary for public health decision-making.

Antipsychotic medications are currently the main pharmacological treatment of schizophrenia [14]. However, some main challenges in the SD management remain, such as the choice among different antipsychotic classes and the patient adherence to the treatment prescribed [15]. Although many schizophrenia patients have the potential to achieve long-term remission and functional recovery under proper treatment, most patients, throughout their life, suffer relapses characterized by an exacerbation of psychosis, which often require emergency room visits and hospitalizations [1, 16–18]. Relapses may also cause subsequent refractoriness to antipsychotic treatment and loss of functional gains [16]. Additionally, and despite clinical practice guidelines for the treatment of schizophrenia recommend the use of monotherapy [12, 19, 20], a wide use of polypharmacy has been described [12].

Understanding the “epidemiological landscape” of SD requires many different types of descriptive studies [8]. Besides sociodemographic aspects, methodological differences, as for example case definition, are also responsible for the observed discrepancies among incidence and prevalence estimations. To our knowledge, there are very few recent studies using Real-World Data (RWD) estimating the incidence and prevalence of schizophrenia [21, 22]. Most studies were not population-based [22] or were only based in hospitalization registers without considering ambulatory care information [21]. The Valencia Region (Spain) has a population of around 5 million inhabitants with universal health coverage and it counts with the Valencia health system Integrated Databases (VID), a healthcare electronic databases network gathering data from hospitalization, primary care, specialist and antipsychotic dispensations, among other [23]. The quality of VID has been shown in several publications [24–30]. The aim of this study was to describe schizophrenia incidence and prevalence, antipsychotic utilization patterns and healthcare utilization (HCU) using the healthcare databases from the Valencia Region, Spain, during an 8-year period (2008–2015).

## Methods

Population-based retrospective descriptive study to estimate prevalence, incidence, and HCU for SD in the Valencia Region, Spain.

### Setting and study population

The Valencia Region, Spain, has a population of approximately five million inhabitants (≈ 10% of the Spanish population) [31]. About 96% of the population is covered by the Regional Health System (RHS) [32]. The RHS is divided into 24 healthcare departments; they include 33 public hospitals, 16 of them attending psychiatric patients, and 241 healthcare districts.

The study population included all individuals aged 15 to 64 years, living in the Valencia Region and insured by the RHS at some point during January 1st, 2008, and December 31st, 2015. Subjects entered the cohort at the start of the study period, on the day of entry into the RHS, or on their fifteenth birthday, whichever occurred last. Follow-up ended when they disenrolled, turned 65 years-old, died, or at the end of the study period, whichever occurred first. Private administrative mutualism and “uninsured” individuals were excluded. For the estimation of healthcare utilization, the cohort of subjects with SD was followed from SD diagnosis until the end of the follow-up.

### Real world data sources: electronic healthcare databases

We used the VID [23] which can be linked through a unique personal identification number [33]. The regional population-based administrative database (SIP) collects and updates demographic data, health services assignment and usage of the health system. The Ambulatory Care Information System (SIA) contains medical information for each patient attended in the outpatient setting (General Practitioners, GPs, and specialist). The minimum basic data set (MBDS) collects all diagnosis and procedures from hospitalizations [34]. Diagnoses were registered using the International Classification of Diseases, Ninth Revision, Clinical Modification coding system (ICD-9-CM) [35]. Drug prescription and dispensation data are registered in the Care Provision Management (GAIA) using the Anatomical Therapeutic Chemical Classification System (ATC); for the purpose of this study, we used only dispensation data.

### Case definitions and study variables

We defined an incident case of SD as the first appearance of an ICD-9-CM code related to schizophrenia spectrum disorders (295.XX) in the outpatient or inpatient (any diagnosis position) settings during the study period.

HCU among patients with SD included outpatient visits to general practitioners, visits to mental health specialists, length of hospital stay and work absences, and antipsychotic dispensations.

Sociodemographic covariates included age, sex, nationality, urban/rural residence, and social exclusion risk (SIP) [36]. Social exclusion risk classification is based on multiple aspects including unemployment, foreign in irregular situation or without resources. Rural areas were classified according to: population density (less than 100 inhabitants per Km^2^), urban nucleus proximity, population trend, percentage of employment in primary, secondary and tertiary sectors, and territorial structure.

We identified antipsychotic drugs through the ATC code N05A and first classified by formulation, differentiating between oral antipsychotic (OA) and long-acting injection (LAI). These two groups were sub-classified into first- and second- generation antipsychotics. Active ingredients included in each of the subgroups are described in Additional file 1. Exposure time to any treatment was defined as time from the first to the last dispensation. If only one dispensation was collected, time was estimated as date of the dispensation plus 30 days. Antipsychotic polypharmacy was defined as the overlap of two or more antipsychotic drugs dispensed simultaneously over time.

### Statistical analysis

Descriptive analyses of baseline sociodemographic characteristics of overall population and population with SD were carried out, including sex, age, nationality, urban status, and social exclusion risk.

Prevalence (total number of patients diagnosed with SD divided by the total number of persons in the study population during the study period) and incidence rates (IR) of SD (total number of incident cases divided by the total number of person-years at risk) were calculated by age, sex and overall. 95% confidence intervals (CI) were calculated by using the Poisson exact method. IR were only calculated in the subpopulation aged between 15 and 34 years. HCU rates (per subject/year) among patients diagnosed with SD were estimated. A descriptive of these results (median and interquartile range, IQR) was reported.

Antipsychotic utilization (dispensations) and polypharmacy patterns among patients with SD were also assessed. Percentage of time (Time %) during which each antipsychotic combination was used by the cohort with SD in the study period was estimated.

Analyses were developed using R Statistical Software version 3.4.3 (Foundation for Statistical Computing, Vienna, Austria).

## Results

### Sociodemographic characteristics of the study population

The study population included 3,976,071 subjects aged 15–64 years (50.7% men and 49.3% women) over the study period. Out of these, a total of 24,749 had a prevalent diagnosis of SD; 15,917 (64.3%) were men and 8832 (35.7%) were women. The majority of subjects with SD were Spaniards (96.4%), urban residents (97.8%), and without social exclusion risk (69.2%).

### Prevalence

Age and sex specific prevalence is shown in Table 1. The overall prevalence (per 1000 persons) for SD was 6.2, having their maximum in adults aged 35–54 years old. SD were 76% more prevalent (7.9 vs. 4.5 per 1000) in men than women.

**Table 1:** Prevalence (per 1000 persons) of SD by age groups and sex (2008–2015)

	Prevalence		
	Cases	Prev.	95% CI
**Age (years)**			
15–24	3090	3.2	(3.1–3.3)
25–34	8542	6.1	(5.9–6.2)
35–44	12,807	8.2	(8.1–8.3)
45–54	11,277	8.8	(8.6–8.9)
55–64	6260	6.2	(6.0–6.3)
**15–64**	**24,749**	**6.2**	**(6.2–6.3)**
**Sex**			
Male	15,917	7.9	(7.8–8.0)
Female	8832	4.5	(4.4–4.6)

*Abbreviations*: *CI* Confidence interval

Prevalence is presented per 1000 persons

**Fig. 1:**
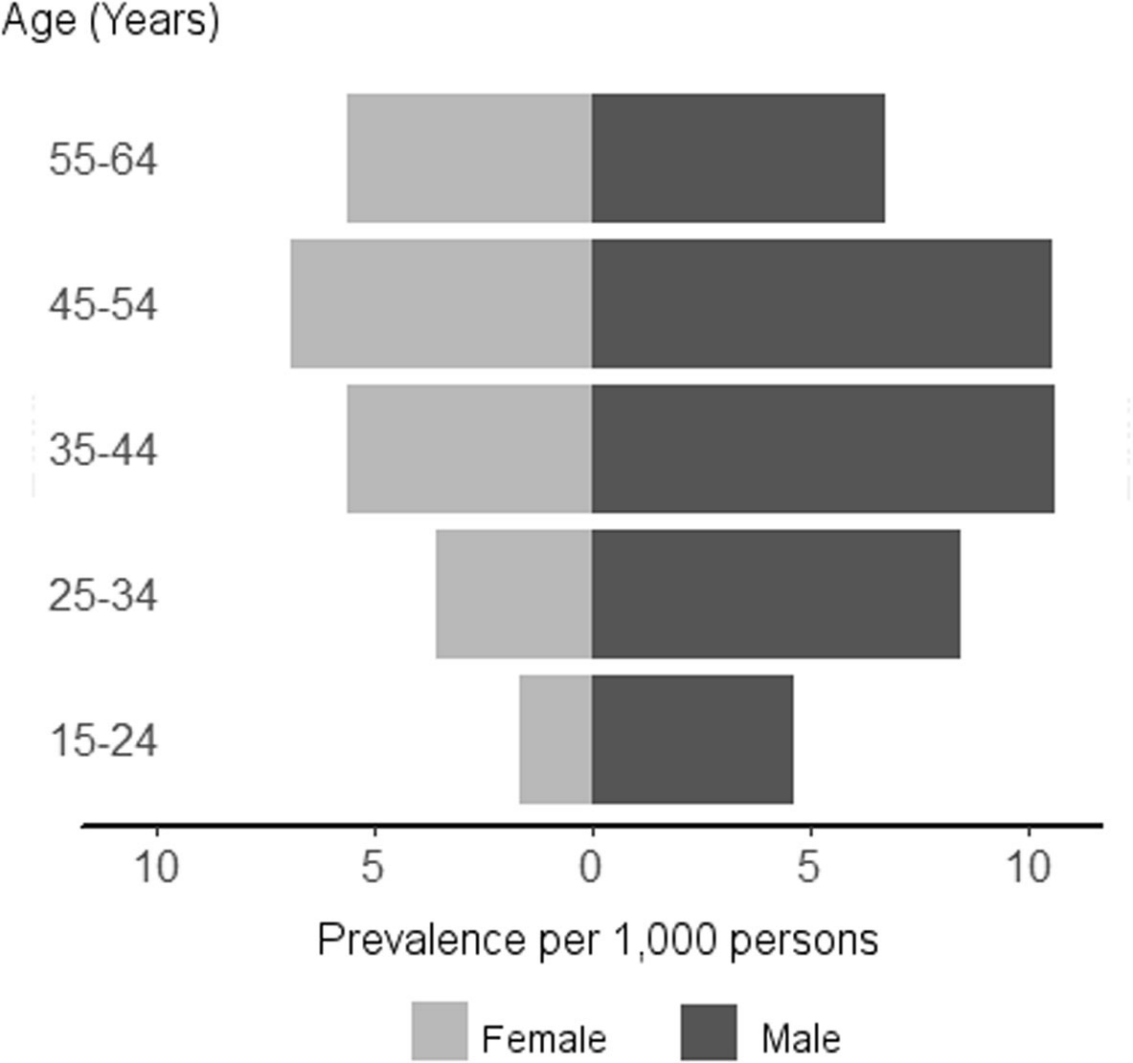
Change in prevalence (per 1000 persons) for schizophrenia by age group and gender (grey for females and dark grey for males) during the study period (2008–2015)

Prevalence by sex and age group is shown in Fig. 1, being higher in men than women in all age groups. The highest prevalence for males was found for the age group 35–54 years (10.6 cases per 1000 persons) while for females the prevalence was highest for the age group 45–54 years (6.9 cases per 1000 persons). Men:women ratio decreased as the age group increased. The biggest differences in sex were found in the youngest groups where prevalence in males was 2.8, and 2.4 higher than in women for the age groups 15–24 and 25–34, respectively.

### Incidence

The overall IR in the subpopulation aged between 15 and 34 years (per 100,000 persons years) was 50.25 (48.86–51.67). The highest incidence was found among the age group of 25–34 years of age with an IR of 57.9 (56.0–59.9). The IR for the group of 15–24 years of age was 39.0 (37.1–41.0). Marked sex differences were found, with overall IR for men (70.64; 68.33–73.00) 2.4 times higher than for women (29.25; 27.75–30.81).

### Healthcare resource utilization (HCU)

Table 2 describes the HCU by the cohort with SD. These subjects resulted in a total of 799,290 outpatient visits for any reason. Of these, 666,347 (83%) were encoded as SD, meaning a median of 3.8 (1.5–7.0) SD visits per person-year. The whole cohort of subjects with SD visited the specialist 312,520 times, corresponding to a median of 0.96 (IQR: 0.0–3.6) specialist visits per person-year. The 21,095 overall hospitalizations with the SD diagnoses code (in any diagnosis position) resulted in 286,139 days of hospital stay with a median of 4 days (IQR: 1.6–9.2) per person-year. Around 10% (2325) of the schizophrenic cohort had any work absence resulting in a total of 292,780 days off sick and a median of 12.5 (IQR: 3.4–28.8) sick days per person-year.

**Table 2:** Healthcare utilization among individuals age 15–64 years diagnosed with schizophrenic disorders: 2008–2015

**Healthcare utilization**	N	Median (IQR)
Overall outpatient visits	799,290	4.5 (2.0–8.2)
Outpatient visits (SD)	666,347	3.8 (1.5–7.0)
^a^ Specialist visits	312,520	0.96 (0.0–3.6)
^b^ Length of hospital stay (days)	286,139	4.0 (1.6–9.2)
^c^ Work absences (days)	292,780	12.5 (3.4–28.8)
Antipsychotics dispensations	2,861,262	15.1 (6.6–28.5)
Antipsychotics dispensations families	N (%)	^d^ Subjects treated (%)
OA-2nd generation	1,696,420 (59.3)	93.4
LAI-2nd generation	576,913 (20.2)	36.0
OA-1st generation	409,121 (14.3)	33.8
LAI-1st generation	106,103 (3.7)	14.7
Other	72,705 (2.5)	13.3

*Abbreviations*: *IQR* Interquartile ratio, *LAI* Long-acting injection antipsychotics, *OA* Oral antipsychotic

Median is presented as a rate in person-year

^a^ Corresponding to mental health specialist visits with the ICD-9-CM code 295.XX

^b^ Corresponding to 21,095 hospitalizations in the cohort of patients with SD

^c^ Corresponding to 5445 work absences in the cohort of patients with SD

^d^ Subjects treated with antipsychotic drugs at some point during the study period

### Utilization patterns of antipsychotics among individuals diagnosed with SD

Among the 24,749 subjects diagnosed with SD, 23,054 (93.2%) were ever treated with some antipsychotic drug during the study period. A total of 2,861,262 dispensations were associated with the treated group, which means a median of 15.1 dispensations per person-year, approximately.

Antipsychotics distribution by formulation and class is shown in Table 2. Almost all, 21,525 (93.4%) out of the 23,054 patients treated, ever used OA-2nd generation during the study period. OA-2nd generation family represented 59.3% of the total dispensations. LAI-2nd generation and OA-1st generation were the next groups of antipsychotics more frequently used with a 36 and 34% of the subjects ever treated with them, respectively. Out of the total dispensations, 20.2 and 14.3% corresponded to LAI-2nd generation and OA-1st generation, respectively. LAI-1st generation were the less commonly used by the schizophrenic cohort (14.7%).

Active ingredients included in each antipsychotic family are described in Additional file 1. The most dispensed active ingredients were risperidone, olanzapine, quetiapine, paliperidone and clozapine, representing 23.8, 12.9, 9.7, 9.7 and 7.2% of the total dispensations studied, respectively.

Out of the 23,054 subjects treated, 29.5% were on treatment with antipsychotic monotherapy, 7.2% were always on two or more antipsychotics while being under treatment (polypharmacy) and 63.3% were on both mono- and polytherapy over the study period. Polypharmacy combinations by antipsychotics formulation and class are described in Table 3. The most prevalent concomitant medications were two or more OA-2nd generation which were ever used by the 38% of the treated subjects. The second and third more frequently used combinations were OA-2nd generation + LAI-2nd generation and OA-1st generation + OA-2nd generation which were used by the 26 and 21.1% of the treated subjects, respectively. Table 3 also shows the percentage of time that each polypharmacy combination has been used during the study period. The combination of two or more OA-2nd generation was used during the longest proportion of time (16.2%), followed by OA-2nd generation + LAI-2nd generation (12.7%). Overall, our cohort with SD was treated with different combinations of polypharmacy during 51.7% of the time.

**Table 3:** Antipsychotic polypharmacy combinations by treatment family in patients diagnosed with schizophrenia: 2008–2015

Polypharmacy combinations	N (% of total)	Subjects %	Time %
OA-2nd generation	18,241 (27.5)	38.1	16.2
OA-2nd generation **+** LAI-2nd generation	16,188 (24.4)	26.0	12.7
OA-1st generation **+** OA-2nd generation	10,271 (15.5)	21.1	7.9
OA-1st generation **+** OA-2nd generation **+** LAI-2nd generation	4213 (6.3)	7.5	2.4
Other^a^ **+** OA-2nd generation	3899 (5.9)	7.9	3.1
LAI-1st generation **+** OA-2nd generation	3325 (5.0)	7.6	3.1
Other combinations	10,281 (15.5)	31.8	6.3

*Abbreviations*: *OA* Oral antipsychotic, *LAI* Long-acting injection antipsychotics

^a^Other family treatments different to OA and LAI

Subjects (%), percentage of subjects ever treated with each polypharmacy combination during the study period

Time (%) represents the percentage of time, of the total study period, during which each combination has been used

For each family, two or more concomitant treatments can be included

## Discussion

This large population-based study provides novel and recent information on the prevalence and incidence of schizophrenic disorders in a southern European region of 5 million inhabitants, and the healthcare resource utilization among these patients using Real World Data from outpatient (primary and specialist care) and inpatient electronic medical records during an 8-year period. In addition, antipsychotic utilization patterns among patients with SD were described focusing on polytherapy, an understudied problem in this population.

The overall prevalence of SD in the region during 2008–2015 was 6.2 per 1000 persons 15–64 years, higher in men than women (men: women rate ratio 1.75). Although differences in estimates used made difficult comparisons, our estimates were within the ranges previously published [3, 7, 8, 37], which reported a lifetime prevalence ranging from 1.6 to 12.1 per 1000 persons. However, our estimated prevalence was greater than the median lifetime prevalence published in prior reviews (4.0–5.5 per 1000) [3, 7, 8]. The differences found could be explained by the heterogeneity and complexity of the disease. In the same line, it has been previously described that the variability in prevalence also depends on the study design, geographic region, case identification methods, study quality, study dates, among others [3, 7]. Moreover, the use of a population-based design using RWD from outpatient (Primary and Specialist care) and inpatient electronic medical records in a setting with universal health coverage might also have minimized the underreporting of prevalent cases.

On the other hand, the overall IR of SD reported in the present study (50.25 per 100,000 persons-year), is higher than the published incidence, ranging from 7.7 to 43.0 per 100,000 persons; median 15.2 [3]. Given that we did not have access to historical data before the study period assessed, it is possible that some cases coded as incidents in people older than 35 years old could have been prevalent, therefore, the IR for the age groups over 35 years old might have been slightly overestimated. Thus, IR calculations were limited to population under 35 years of age. Although this limitation would be of low impact as compared with studies using claims, given that when using electronic records if a chronic diagnosis is registered this remains active lifelong or until it is resolved. Nevertheless, the estimated IR for the age groups from 15 to 34 years old can be considered highly reliable due to the onset of symptoms usually occurs during the second and third decades of life [4, 5]. Furthermore, the IR observed in these age groups are aligned with a recent study from Denmark using population-based data from 2000 to 2012 [21]. In any case, to collect incidence data for all age groups allowed us to make a better estimate of the prevalence.

Concerning sex differences, we found a 60 and 75% higher prevalence and incidence rates in males than females, respectively. While there is a tacit understanding that sex differences in the incidence of schizophrenia are robust [3, 21, 37], several reviews did not find gender differences in prevalence [3, 8, 9, 38, 39]. From a statistical point of view these discrepancies between incidence and prevalence are unexpected [3, 8], being reasonable to observe higher prevalence in men as found in the incidence rates. Few studies have shown the expected sex differences in prevalence [22]. Some possible explanations for these differences have been suggested, as for example differences in the course of illness that have been demonstrated to be more severe in men than in women [8], implying higher utilization of health care services and, therefore, increasing the probability of detecting SD cases.

The difference in prevalence by gender was observed in all age groups, but the male: female ratio decreased as the age increased. These findings are in line with previous evidence showing that the age of onset in schizophrenia varies between males and females, where males tend to have an earlier onset [37]. Other explanations suggest that more women than men have onset later in life due to the declining of the protective effect exerted by estrogens [40] or due to psychosocial precipitating factors [41].

Despite the low prevalence of schizophrenia, the individual and societal burden associated with the disorder is very high in terms of mortality, morbidity, and economic and social costs. Population-based studies on healthcare resource utilization (HCU) are scarce and some of the most recent are focused on antipsychotic treatments patterns [10, 12, 13, 42]. In addition, previous studies on HCU for SD have shown great variability among sites [43]. The present study provides a descriptive of the HCU, including primary care and specialist’s visits, hospitalizations, work absences and antipsychotics dispensations during a recent 8-year period in a cohort of almost 25,000 patients with SD. Our cohort of patients with SD visited the General Practitioner (GP) a median of 4.5 times annually, and 3.8 of these visits (83.4%) were related to SD. Regarding hospitalizations, the median LOS of hospitalizations encoded with a SD diagnosis associated (21095) was 4 days, whereas our average LOS was 10 days (data not shown). Although these results are comparable to other European studies [43–45], the average LOS of acute psychotic episodes may be longer [46]. The fact that we considered hospitalizations for SD in any diagnosis position could have influenced these estimates. On the other hand, it has been shown a median of 12.5 work absence days per patient-year. However, these figures should be interpreted with caution as it has been estimated that three out of four patients with schizophrenia have dropped out of the job market [47].

High quality recent studies focused on the analysis of antipsychotic prescriptions patterns [10, 12, 13, 42]. Regardless this was not our main objective; we have performed a cursory description of the antipsychotic dispensation in our large cohort of patients (24749) with SD. In agreement with other literature [12, 42, 48], OA-2nd generation was the principal treatment for most of our patients. It is also important to highlight that, contrary to recommended guidelines [48] and in alignment with previous studies in other region of Spain [12], the majority of our patients (around 70%) were ever treated with antipsychotic polypharmacy.

On the other hand, an interesting result that can be found in this study is the percentage of subjects with treatment-resistant schizophrenia. This information could be estimated by reporting the proportion of subjects treated with clozapine [49]. In this study, this proportion represented the7.2% of the total antipsychotics dispensations accounted. This information would be very useful, since a significant amount (30–60%) of individuals with schizophrenia fail to respond to standard treatments with antipsychotics [50].

The main strength of this study is the use of the electronic healthcare databases which reflects real-life practice on a population of around 5Mill subjects with universal (96%) health coverage. The quality of these databases has been widely demonstrated in previous studies on epidemiology, treatment patterns, resource use, and drug safety and effectiveness [24–27, 30, 35, 51]. As we already pointed out, the use of multiple clinical sources of information not only from mental health services, but also from primary care, specialists, and hospitalizations confers reliability to the study. Nevertheless, some limitations should be taken into account. First of all, historical data before 2008 were not available so, as mentioned above, the IR showed for the age group of over 35 years old might be overestimated. Secondly, our polypharmacy definition could have been biased as no gaps between concomitant treatments were considered. However, as shown in Table 3, percentage of time that each polypharmacy combination was used has been described.

HCU and antipsychotics dispensations patterns data described in this study constitutes a cornerstone for future deeper population-based studies on SD including time-variations on antipsychotics prescriptions and dispensations, treatments adherence and costs.

## Conclusions

This large population-based study using RWD provides novel and recent information SD in a southern European country. The prevalence and IR of SD showed is greater than previously published and higher in men than in women. The fact of having used a large arsenal of electronic data (including outpatient and inpatient) for 8 years may have influenced. SD represents high burden and HCU. Contrary to guidelines recommendations the majority of patients were ever treated with antipsychotic polypharmacy.

## Supplementary information

**Additional file 1.** Including information about all active ingredients included in each antipsychotic family (LAI First-Generation, OA First-Generation, LAI Second-Generation and OA Second-Generation).

## Data Availability

The dataset analyzed (dissociated and aggregated) during the current study are available in this repository: https://drive.google.com/file/d/12iBuhTowzf3I1Jc08HKxm7sBFVrDKpPm/view?usp=sharing.

## Abbreviations

ATC: Anatomical Therapeutic Chemical Classification System
CI: Confidence intervals
GAIA: The Care Provision Management
HCU: Healthcare utilization
IQR: Interquartile range
IR: Incidence rate
LAI: Long-acting injection
MBDS: Minimum basic data set
OA: Oral antipsychotic
RHS: Regional Health System
RWD: Real-World Data
SIP: Population-based administrative database
SD: Schizophrenia spectrum disorders
VID: The Valencia health system Integrated Databases
