# Supplementary material for: Epidemiology of schizophrenia and its management over 8-years period using real-world data in Spain
Source: BMC Psychiatry. 2020 Apr 5;20:149. doi: 10.1186/s12888-020-02538-8 (PMC7132863; doi:10.1186/s12888-020-02538-8)
Supplement: Supplementary file 1 — Additional file 1. Including information about all active ingredients included in each antipsychotic family (LAI First-Generation, OA First-Generation, LAI Second-Generation and OA Second-Generation). [file 12888_2020_2538_MOESM1_ESM.docx]

**Additional file 1. Active ingredients included in each antipsychotic family.**

- - - - **LAI First-Generation**
  - Fluphenazine
  - Haloperidol (inyectable)
  - Pipotiazine
  - Zuclopenthixol (inyectable)
    - - **OA First Generation**
  - Perphenazine
  - Haloperidol
  - Chlorpromazine
  - Pericyazine
  - Pimozide
  - Sulpiride
  - Flupenthixol
  - Zuclopenthixol
  - Levomepromazine
    - - **OA Second Generation**
  - Risperidone
  - Paliperidone
  - Aripiprazole
  - Olanzapine
  - Clozapine
  - Amisulpride
  - Clotiapine
  - Quetiapine
  - Sertindole
  - Ziprasidone
    - - **LAI Second Generation**
  - Paliperidone palmitate
  - Aripiprazole
  - Risperidone
  - Olanzapine Pamoate
    - - **Other: any other antipsychotic not included above**
